# The number of fusion levels as a potential factor influencing long-term complications of anterior controllable antedisplacement fusion: a biomechanical analysis

**DOI:** 10.3389/fsurg.2026.1800944

**Published:** 2026-04-29

**Authors:** Gaole He, Haopeng Li, Liang Yan, Zhongkai Liu, Teng Lu

**Affiliations:** 1 Health Science Center, Xi'an Jiaotong University, Xi'an, Shaanxi, China; 2 Department of Spine Surgery, Honghui Hospital, Xi'an Jiaotong University, Xi'an, Shaanxi, China; 3 Department of Spine and Bone Tumor Surgery, Xi'an Jiaotong University Second Affiliated Hospital, Xi'an, Shaanxi, China

**Keywords:** adjacent segment degeneration, anterior controllable antedisplacement fusion, cervical fusion levels, complications, finite element analysis

## Abstract

**Background:**

Anterior controllable antedisplacement fusion (ACAF) is widely used for cervical ossification of the posterior longitudinal ligament, but long-term complications, such as adjacent segment degeneration (ASD), pseudarthrosis, cage subsidence, and implant failure, remain nonnegligible. This study aimed to explore the influence of the number of fusion levels (NFL) on these complications through finite element (FE) analysis, providing a biomechanical basis for optimizing surgical strategies for ACAF.

**Methods:**

Three FE ACAF models (two-level, three-level, and four-level) were established on the basis of a validated C2–T1 cervical spine model. A hybrid loading protocol with a 75 N follower load and physiological moments was applied to simulate physiological motions. Key parameters, including the range of motion (ROM) of the surgical and adjacent segments, disc stress, facet joint force (FJF), endplate stress, and the plate, screw, and screw–bone interface stresses, were compared among the three models.

**Results:**

An increase in the NFL led to significant increases in the ROM, disc stress, and FJF of adjacent segments, with the upper adjacent segment showing more prominent changes than the lower segment. The ROM of the surgical segment gradually increased with increasing NFL, and the fusion space micromotion correspondingly increased. Endplate stress and implant-related stresses (plate, screw, and screw–bone interface stresses) all tended to increase steadily with increasing NFL, reflecting a continuous increase in the mechanical load at the surgical site and in the adjacent segments.

**Conclusions:**

The NFL is a potential risk factor for long-term complications of ACAF. An increase in the NFL raises the mechanical load in the surgical and adjacent segments, thereby potentially increasing the risks of ASD, pseudarthrosis, cage subsidence, and implant failure.

## Background

Anterior controllable antedisplacement fusion (ACAF), or vertebral body sliding osteotomy, is promising technique for treating cervical ossification of the posterior longitudinal ligament (OPLL) (1–3). It improves upon traditional decompression methods by dissociating and anteriorly translating the vertebral body–OPLL complex, resulting in effective spinal canal decompression without the need for direct OPLL resection (4). In recent years, numerous clinical studies have confirmed the advantages of ACAF over conventional surgical techniques, such as anterior cervical corpectomy and fusion (ACCF) and laminoplasty, including superior recovery of neurological function, more robust restoration of cervical lordosis and the spinal canal space, and a lower incidence of complications (1, 3, 5).

Nevertheless, despite these benefits, ACAF still results in a variety of long-term complications that can negatively affect surgical outcomes (5, 6). In anterior cervical fusion surgeries, the most common long-term complications include cage subsidence, implant failure, pseudarthrosis, and adjacent segment degeneration (ASD) (7–9). For ACAF specifically, the reported incidences of these complications are 5.5–14.3% for cage subsidence, 2.8–4.1% for screw breakage or loosening, and 2.8–7.1% for pseudarthrosis (5, 6). Notably, although these values are lower than those observed for ACCF, they remain substantial (5). Therefore, clarifying the etiological factors influencing the development of long-term complications in ACAF is of paramount clinical importance, as it would enable the development of targeted preventive strategies to reduce the risk of complications.

The number of fusion levels (NFL) may be a potential factor influencing the incidence of long-term complications in ACAF because quantity theoretically affects the biomechanical properties of the cervical spine, which in turn alters the distributions of motion and stress in both the surgical and adjacent segments (10–12). However, the relevance of the NFL to long-term complications following ACAF has not been investigated. We conducted a finite element (FE) analysis based on three ACAF FE models with different NFLs to address this gap, aiming to determine whether the NFL is a potential factor influencing long-term complications following ACAF. Specifically, the range of motion (ROM) of the surgical segment was assessed to evaluate the potential role of the NFL in pseudarthrosis; the ROM, disc stress, and facet joint force (FJF) of the adjacent segment were assessed to assess the potential association between the NFL and the ASD; the endplate stress was assessed to determine the potential impact of the NFL on cage subsidence; and the stresses at the plate, screw, and screw–bone interface were assessed to explore the potential influence of the NFL on implant failure.

## Methods

### Intact C2–T1 FE model

Our previously validated FE model of the C2–T1 cervical spine was used to establish the ACAF constructs (13). For this intact model, convergence analysis was performed, in which the maximum variations in both the disc strain energy and the FJF were controlled to be less than 5% (13). Additionally, this FE model had been successfully validated by comparing the ROM and FJF with relevant data from other published studies (14–20). Detailed specifications regarding the geometries, element types, element counts, and material properties of the tissue components within the C2–T1 FE model have been documented in our previous publication and thus are not reiterated here (13).

### FE modeling of the ACAF constructs

**Figure 1:**
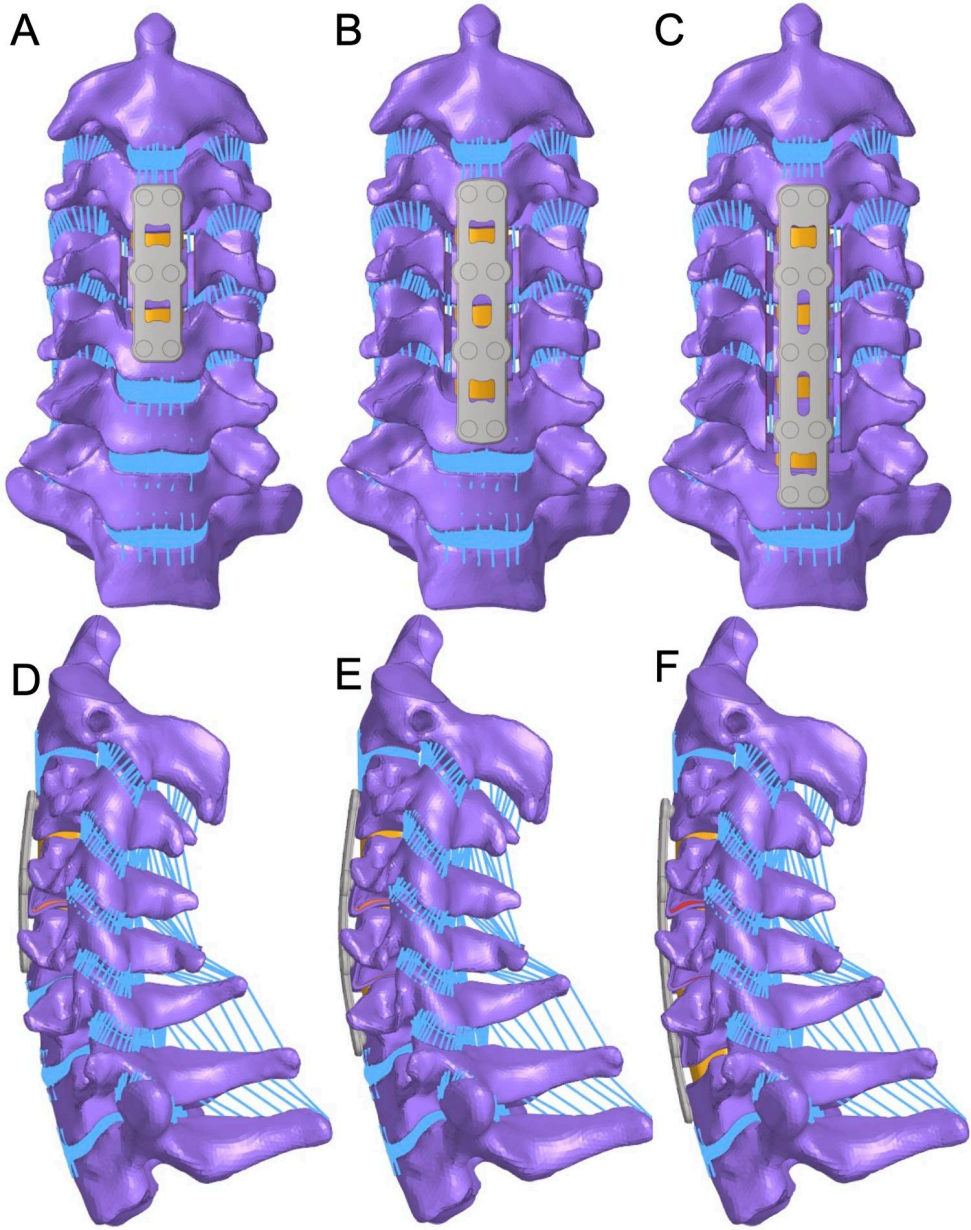
(**A,D**) Two-level (C3–5), (**B,E**) three-level (C3–6), and (**C,F**) four-level (C3–7) ACAF FE models.

In total, three ACAF constructs with different NFLs were modeled, including two-level (C3–5), three-level (C3–6), and four-level (C3–7) ACAF models (Figure 1). The modeling of the three ACAF constructs adhered to a consistent core workflow (12, 21). In the initial step of tissue resection, intervertebral discs were removed from the predefined target levels. For the two-level procedure, the discs of C3/4 and C4/5 were excised. For the three-level procedure, the discs of C3/4, C4/5, and C5/6 were removed. For the four-level procedure, the discs of C3/4, C4/5, C5/6, and C6/7 were resected. Concurrently, the anterior and posterior longitudinal ligaments were dissected over the level ranges corresponding to each procedure type: C3–C5 for the two-level procedure, C3–C6 for the three-level procedure, and C3–C7 for the four-level procedure (12, 21).

Following tissue resection, the anterior portion of the vertebral body at an average thickness of was removed (12, 21). For the two-level procedure, this resection was limited to the C4 vertebral body; for the three-level procedure, it was extended to the C4 and C5 vertebral bodies; and for the four-level procedure, it included the C4, C5, and C6 vertebral bodies. After vertebral body resection, implant placement and fixation were performed. Bone graft-filled interbody cages were inserted into each target disc space, with the number of cages matching the number of target levels. Anterior plates were then fixed to the superior and inferior vertebral bodies of the level range of each procedure using four self-tapping screws per plate.

Next, bilateral grooves with a thickness of 3 mm were created on the medial side of the uncinate processes of the intermediate vertebral bodies (12, 21). The locations of these grooves aligned with the vertebral bodies subjected to anterior resection: the C4 vertebral body for the two-level procedure; the C4 and C5 vertebral bodies for the three-level procedure; and the C4, C5, and C6 vertebral bodies for the four-level procedure. Finally, the intermediate vertebral bodies were pulled ventrally, and the middle self-tapping screws of the anterior plate were tightened to secure the construct. The transverse and anteroposterior diameters of the cage were 14 mm and 12 mm, respectively. The diameter and length of the self-tapping screw were 3.5 mm and 16 mm, respectively. The width of the plate was 16 mm, and the lengths were 48 mm, 70 mm, and 85 mm for the two-level, three-level, and four-level ACAF models, respectively. A “tie” constraint was assigned to the cage–endplate, screw–bone, and screw–plate interfaces to simulate rigid fixation conditions. The element types and material properties of the implants are shown in Table 1.

**Table 1:** Element type and materials properties assigned to the implants.

Material	Element type	Elastic modulus (MPa)	Poisson ratio
Cage (Polyetheretherketone)	C3D4	3,760	0.37
Plate (titanium alloy)	C3D4	110,000	0.34
Screw (titanium alloy)	C3D4	110,000	0.34
Bone graft (cancellous bone)	C3D4	Neo-Hookean (*C*_10_ = 19.38, *D* = 0.0252)	

### Loading conditions

The hybrid loading protocol proposed by Panjabi et al. (15) was employed (22). For all three ACAF FE models, the interior surface of the T1 vertebra was fully constrained in all directions. A follower load of 75 N was imposed on the superior surface of the C2 vertebra to replace the action of head gravity and neck muscle force and allow the FE model to more closely approximate the natural function of neck muscles (13, 23). For the four-level ACAF model, a physiological moment of 1.5 Nm, which is close to the torque range borne by the cervical spine during actual daily activities, was applied to C2 to simulate extension, flexion, lateral bending, and axial rotation (13). The two-level and three-level ACAF FE models were subsequently moved under moment control until they reached the same ROM as the four-level ACAF model. The two-level ACAF model required moments of 0.58 Nm, 0.49 Nm, 0.72 Nm, and 0.41 Nm for extension, flexion, lateral bending, and axial rotation, respectively, to match the ROM of the four-level ACAF FE model. In contrast, the three-level ACAF model needed higher moments of 0.79 Nm, 0.85 Nm, 0.97 Nm, and 0.66 Nm for the same motion directions. This loading design realistically simulates a standardized *in vitro* experimental scenario in which the total ROMs of different models are comparable, enabling the capture of adaptive compensatory movements and biomechanical changes in adjacent and surgical segments (22).

### Data extraction

After the FE analysis, the ROMs at the surgical and adjacent segments, the FJF at the adjacent segments, and the mean stresses of the endplate, fixation plate, screw, screw–bone interface, and adjacent disc were obtained and compared across the three ACAF models.

## Results

### Model validation of the ACAF model

**Figure 2:**
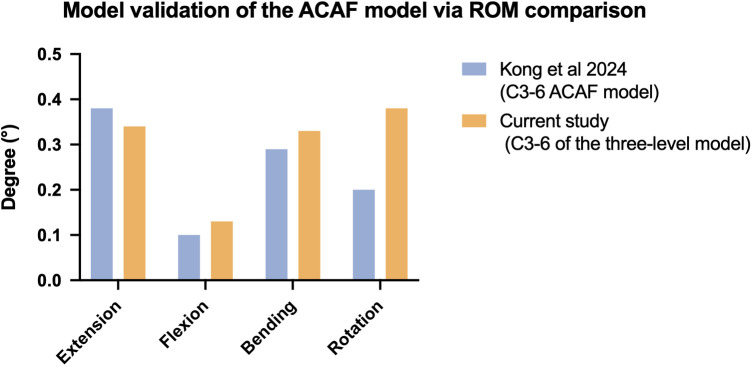
Model validation of the ACAF model via ROM comparison.

To validate the biomechanical reliability of the constructed FE models, the ROM of the three-level ACAF construct (C3–6) in the current study was compared with the published FE data of Kong et al. (21) across four physiological motion planes (Figure 2). The results demonstrated a strong correlation and numerical consistency between the two studies. In detail, the ROM values for extension, flexion, lateral bending, and axial rotation in our model were 0.34°, 0.13°, 0.33°, and 0.38°, respectively. These values closely matched the corresponding ROMs reported by Kong et al. (0.38°, 0.10°, 0.29°, and 0.20°) (21). The minimal discrepancies in the quantitative biomechanical responses confirm the validity of our FE modeling methodology and parameter assignments, thereby establishing a solid foundation for the subsequent analysis of fusion level effects.

### ROM

**Figure 3:**
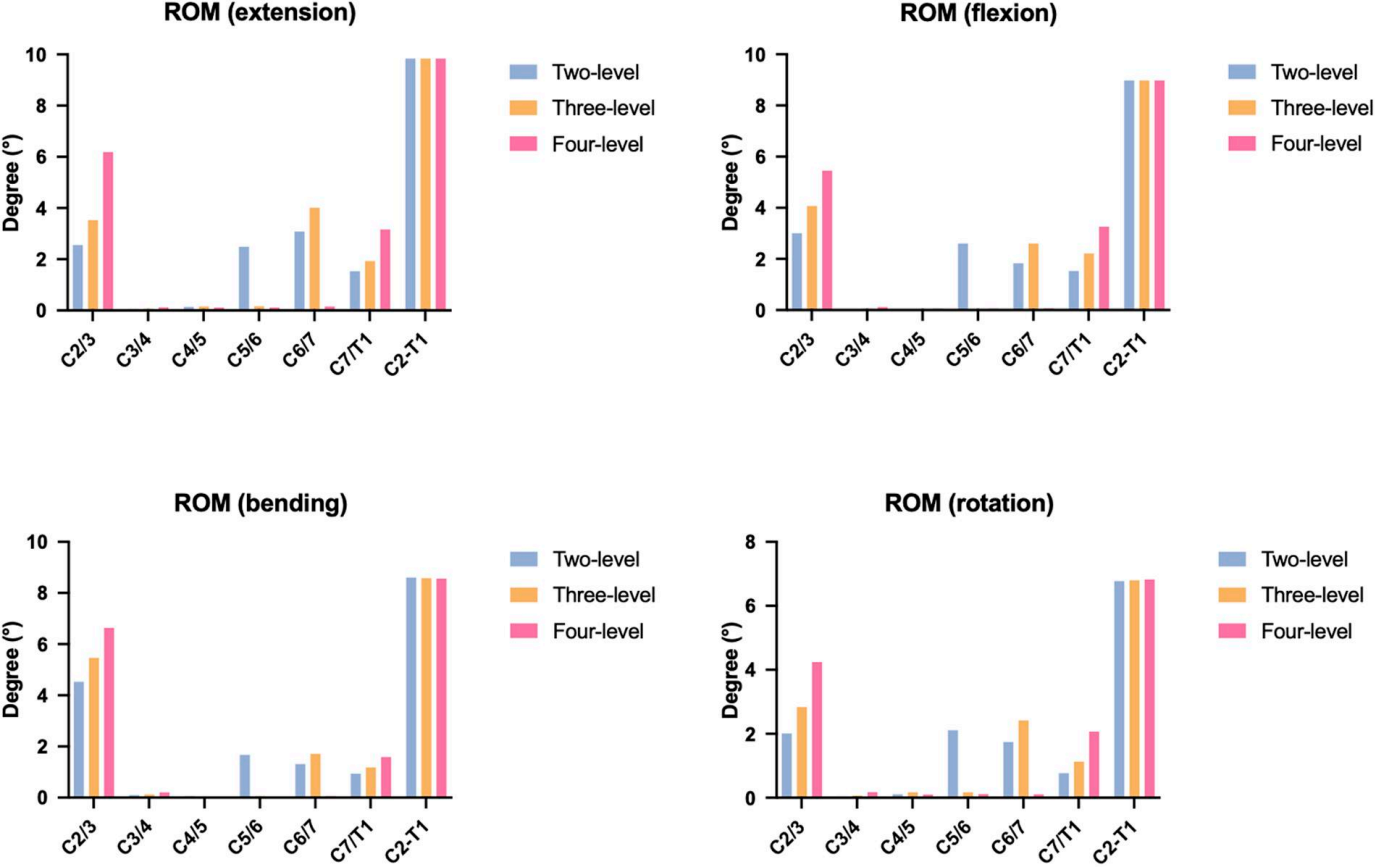
Total and segmental ROMs.

The ROMs of the three ACAF models are shown in Figure 3. All the models exhibited identical total ROMs across different movements, with values of 9.84° for extension, 8.98° for flexion, 8.58° for lateral bending, and 6.8° for axial rotation. A clear trend emerged in the ROM distribution between surgical and adjacent segments as the NFL increased. For the two-level ACAF model, the surgical segment had ROMs ranging from 0.03° to 0.19° in all directions; this range expanded slightly to 0.1°–0.41° for the surgical segment of the three-level model and to 0.29°–0.51° for the surgical segment of the four-level model, indicating a gradual increase in surgical segment mobility with additional fusion levels.

In contrast, the adjacent segment ROM showed a more pronounced upward trend, especially in the upper adjacent segment, with the NFL. The upper adjacent segment of the two-level model had ROMs of 2.01–4.35°, which increased to 2.84–5.46° in the three-level model and reached 4.24–6.64° in the four-level model. The lower adjacent segments also followed this pattern, but with a smaller magnitude. The lower segments of the two-level, three-level, and four-level models had ROMs of 1.67°–2.49°, 1.71°–4.01°, and 1.59°–3.16°, respectively, reflecting the greater motion compensation among the upper adjacent segments than among the lower segments did as the number of fusion levels increased.

### Mean disc stress

**Figure 4:**
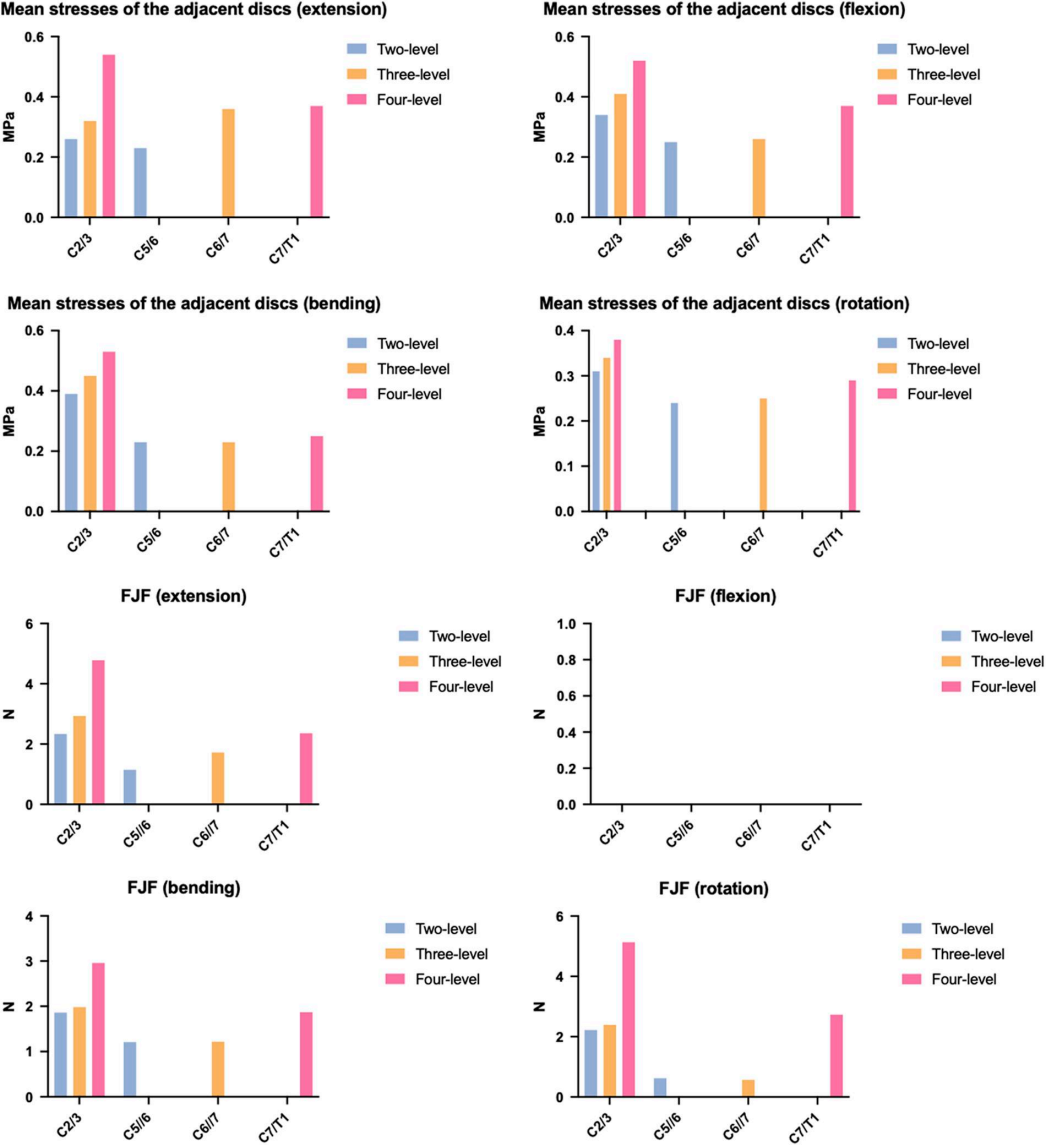
FJFs of the adjacent segments and mean stresses of the adjacent discs.

**Figure 5:**
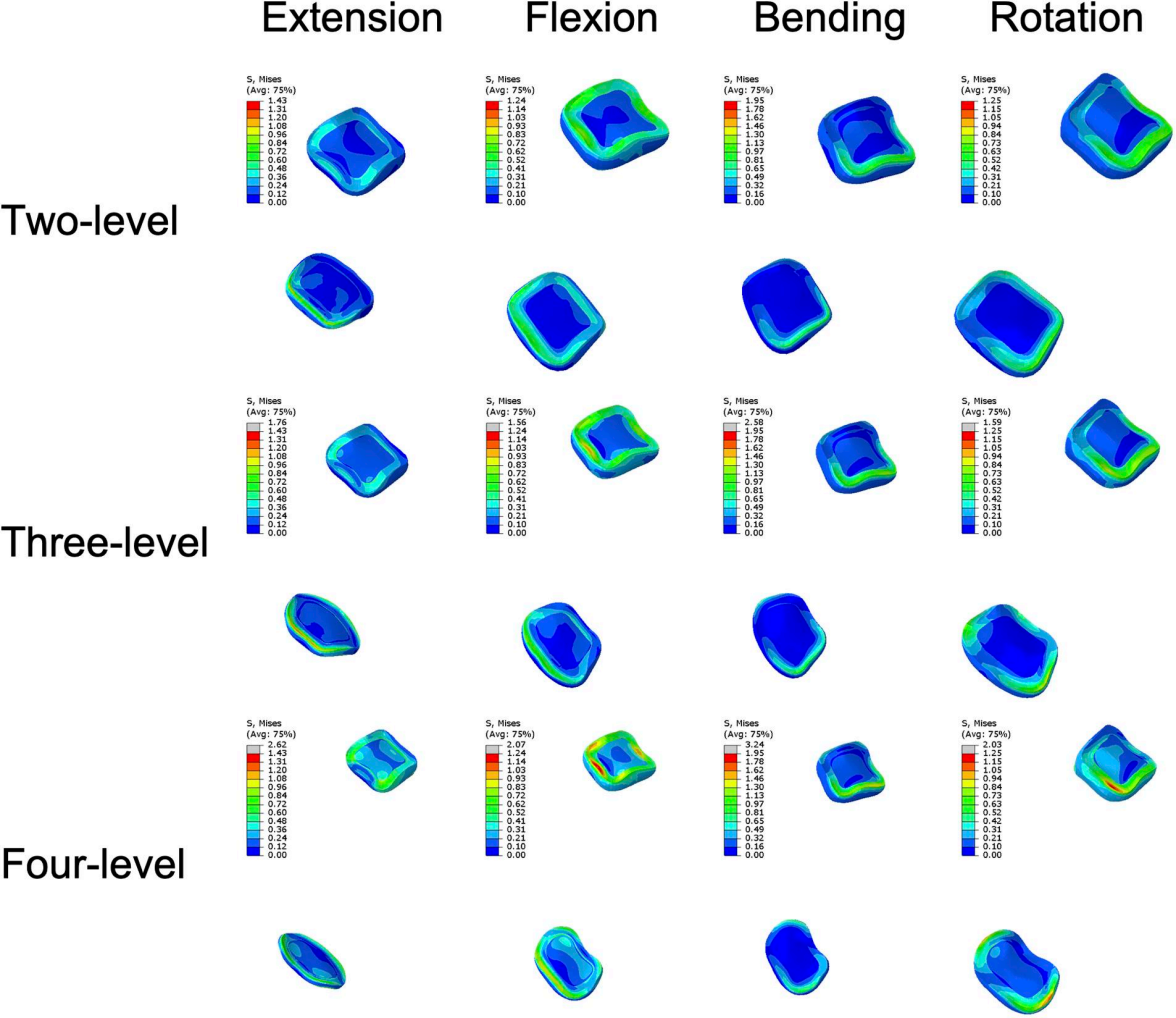
Stress contour plots of the adjacent discs.

The mean disc stresses of the adjacent segments are presented in Figure 4, which shows consistent increases as the NFL increased. Similar to the ROMs, these increases were most notable in the upper adjacent segments. For the upper adjacent segments, the two-level model exhibited a mean disc stress range of 0.26 MPa to 0.39 MPa; this range expanded to 0.32 MPa to 0.45 MPa in the three-level model and to 0.38 MPa to 0.54 MPa in the four-level model. The lower adjacent segments showed a weaker increasing trend in mean disc stress. The two-level model had a mean disc stress range of 0.23 MPa to 0.25 MPa, whereas the value of the three-level model ranged from 0.23 MPa to 0.36 MPa, and the value of the four-level ACAF model ranged from 0.25 MPa to 0.37 MPa. The stress contour plots of the adjacent discs (Figure 5) visually confirmed this increasing stress distribution.​

### FJF

The FJF of the adjacent segments is also shown in Figure 4, with a consistent increasing trend as the NFL increased. More prominent changes occurred in the upper adjacent segments. For the upper adjacent segment, the two-level model had an FJF ranging from 0 N to 2.34 N. This range expanded to 0 N to 2.93 N in the three-level model and further to 0 N to 5.13 N in the four-level model. The lower adjacent segments exhibited a less pronounced increasing trend in the FJF. The two-level model had an FJF of 0 N to 1.21 N at C5/6, the three-level ACAF model had an FJF of 0 N to 1.72 N at C6/7, and the four-level model had an FJF of 0 N to 2.73 N at C7/T1.

### Endplate stress

**Figure 6:**
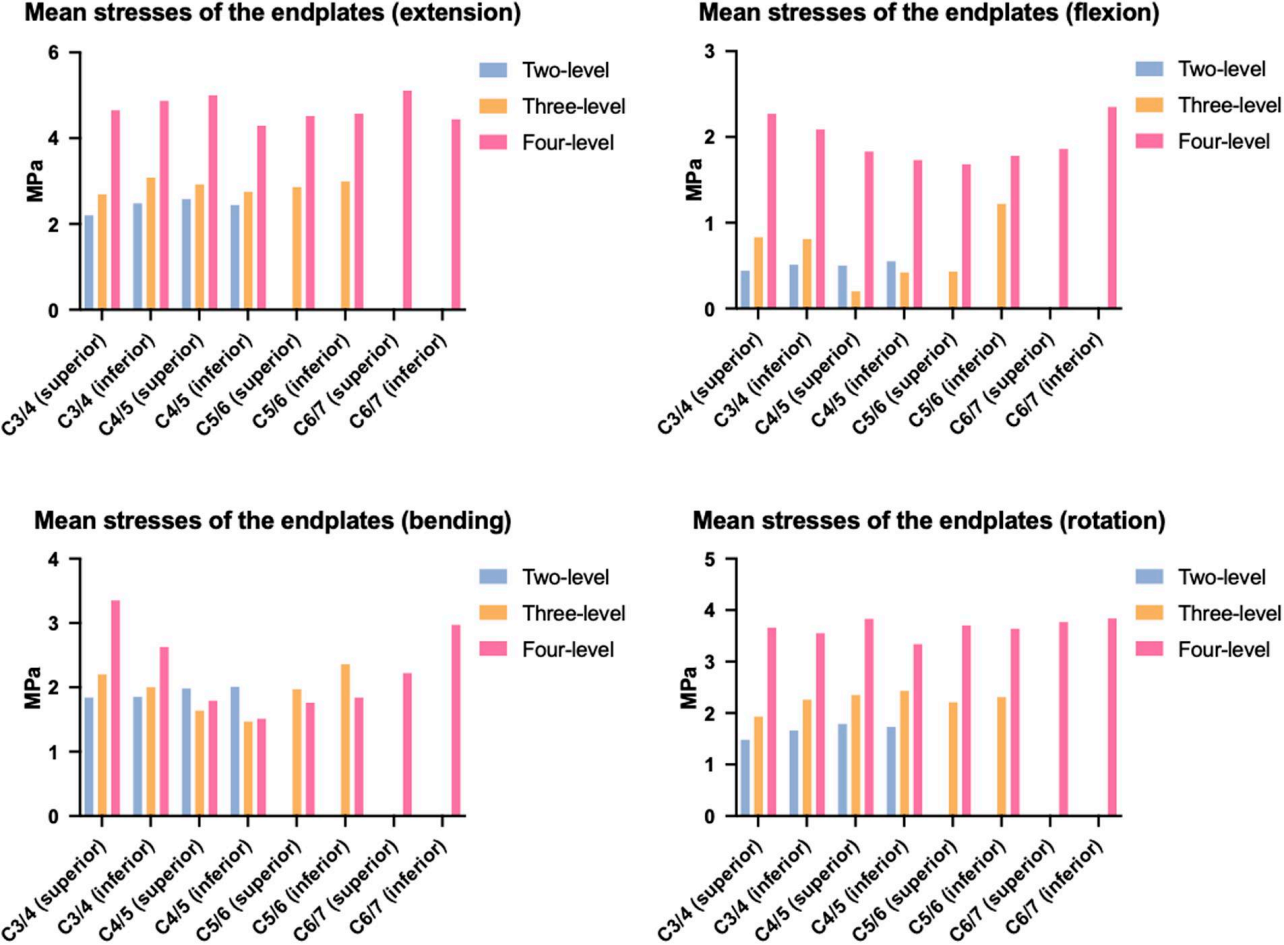
Mean stresses of the endplates of the surgical segments.

**Figure 7:**
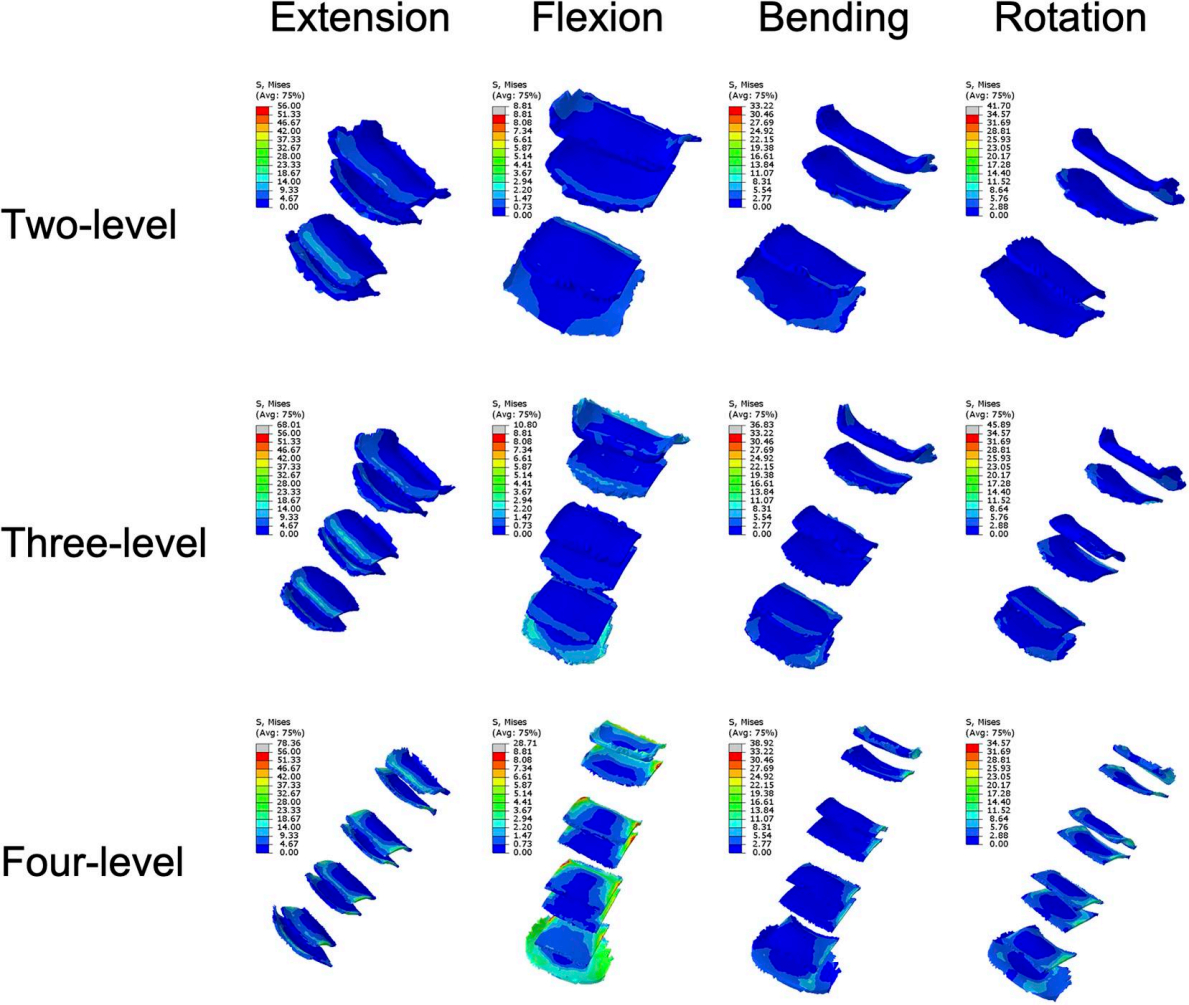
Stress contour plots of the endplates of the surgical segments.

The mean endplate stresses of the three ACAF models are displayed in Figure 6, with distinct trend differences across fusion levels. The mean endplate stress of the two-level model ranged from 0.44 MPa to 2.58 MPa, while that of the three-level model ranged from 0.43 MPa to 3.08 MPa. In contrast, the mean endplate stress of the four-level model sharply increased, with a range of 1.51 MPa to 5.11 MPa. The stress contour plots of the endplates are shown in Figure 7.

### Implant stresses

**Figure 8:**
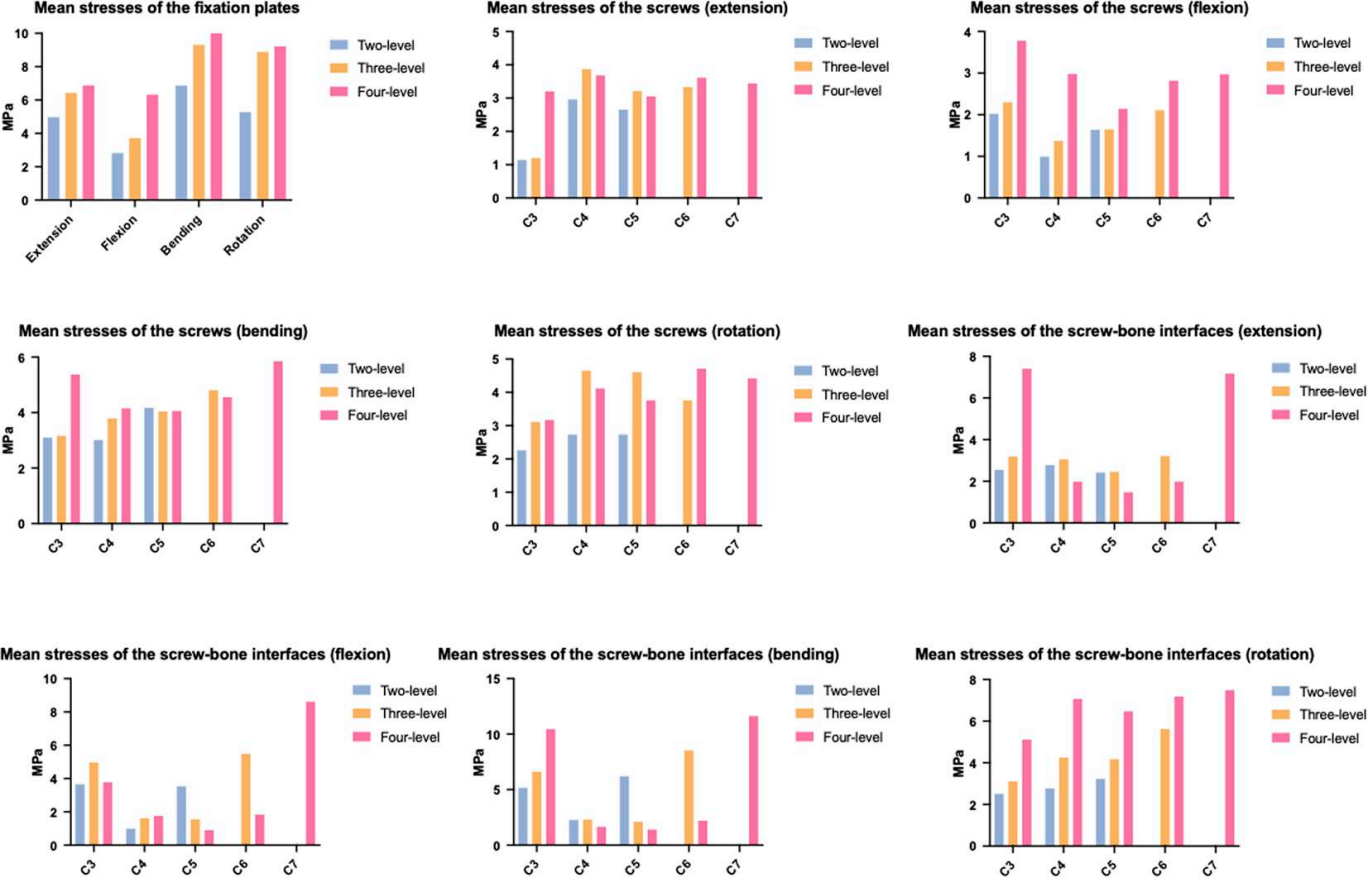
Mean stresses of the plates, screws, and screw–bone interfaces.

**Figure 9:**
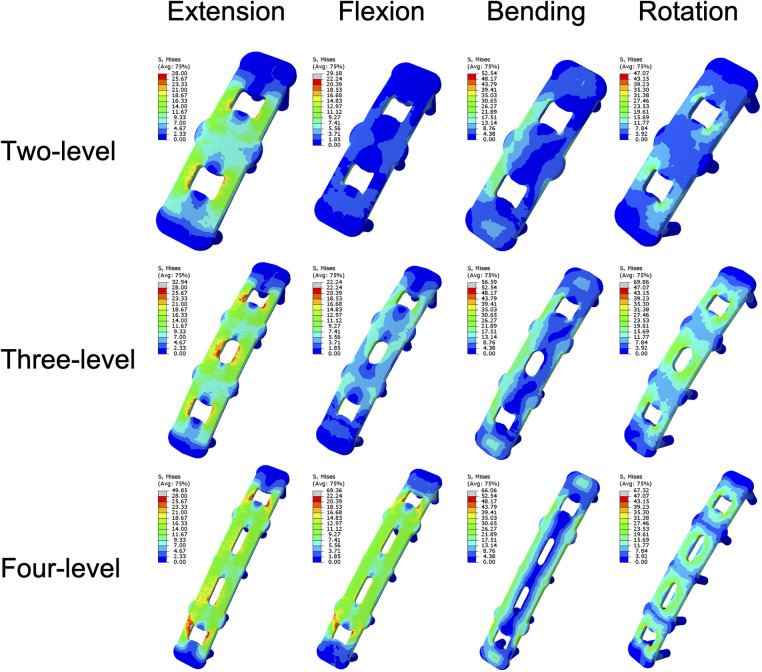
Stress contour plots of the screw–plate systems.

**Figure 10:**
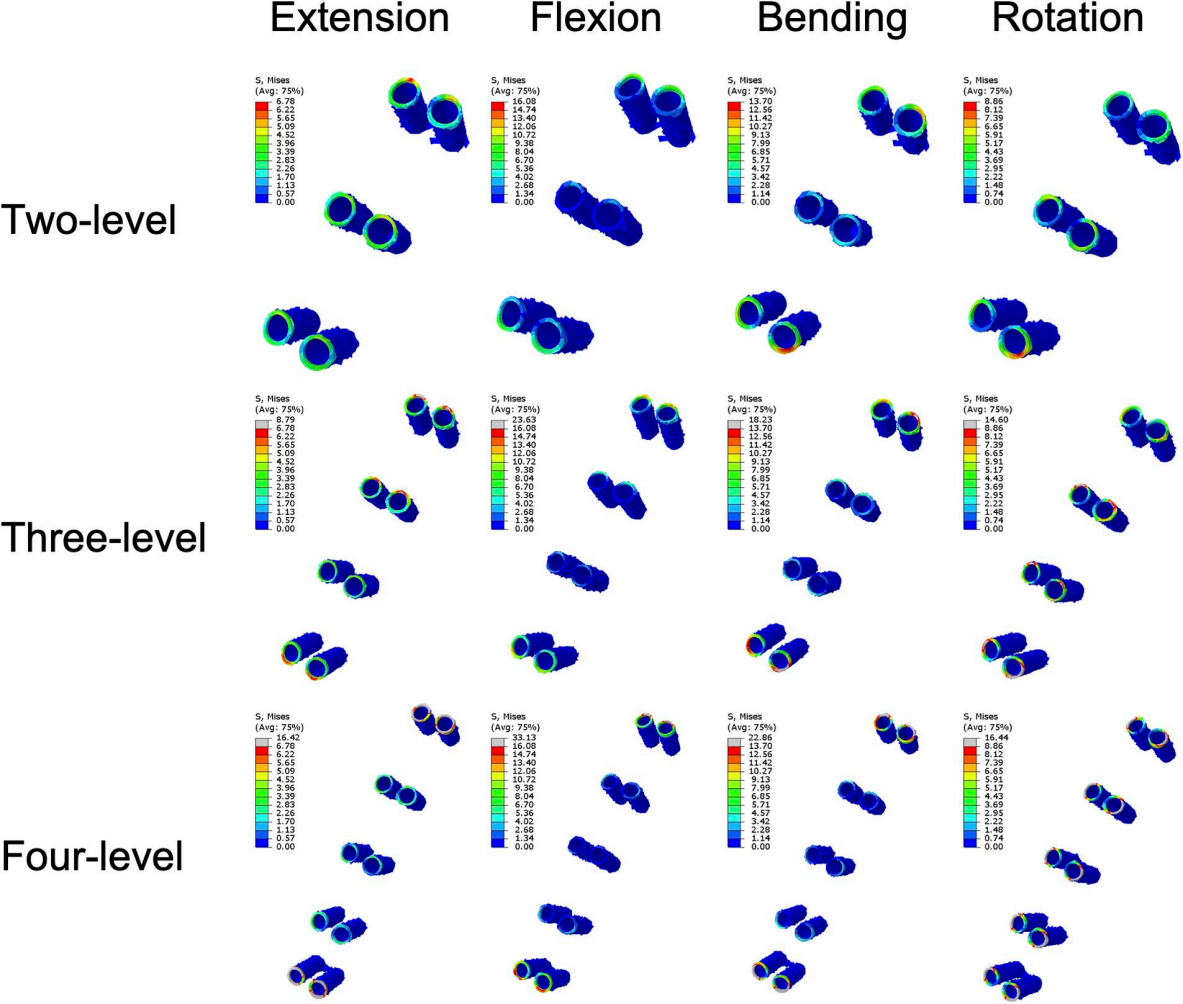
Stress contour plots of the screw-bone interfaces.

The stress on the implants tended to increase steadily with increasing NFL across all the components, reflecting a greater mechanical load on the implants (Figure 8). The mean plate stress of the two-level model ranged from 2.82 MPa to 6.87 MPa, that of the three-level model ranged from 3.72 MPa to 9.31 MPa, and that of the four-level model ranged from 6.32 MPa to 12.09 MPa (Figure 9). The mean screw stress showed a similar upward pattern; the stress of the two-level model ranged from 0.99 MPa to 4.17 MPa, that of the three-level ACAF model ranged from 1.2 MPa to 4.81 MPa, and that of the four-level model ranged from 2.14 MPa to 5.85 MPa (Figure 8). The mean stress at the screw–bone interface of the two-level model ranged from 0.99 MPa to 6.2 MPa, that of the three-level model ranged from 1.55 MPa to 8.52 MPa, and that of the four-level model ranged from 0.9 MPa to 11.62 MPa (Figure 8). The stress contour plots of the plates, screws, and screw–bone interfaces further illustrate this increasing stress pattern (Figures 9, 10).

## Discussion

The findings of the present FE study indicate that as the NFL in ACAF increases, the ROM, mean intervertebral disc stress, and FJF in both the upper and lower adjacent segments significantly increase, which may accelerate the development of ASD. Concurrently, the ROM of the surgical segment also increases, which in turn increases micromotion within the intervertebral space designated for fusion and thus increases the risk of pseudarthrosis. Furthermore, an increase in the NFL leads to greater stress magnitudes in the endplate, screw–plate construct, and screw–bone interface, subsequently increasing the risks of cage subsidence and implant failure. Collectively, these results demonstrate that the NFL represents a potential risk factor for long-term complications following ACAF.

### Adjacent segment

Our results reveal for the first time the potential association between the NFL and the risk of ASD following the ACAF procedure. As the NFL increased from two to four, the ROMs in the upper adjacent segment increased by 52.6%–109.5%, and the mean disc stress and FJF increased by 38.46%–46.15% and 0%–116.5%, respectively. The primary cause of these changes is that a cervical spine with a higher NFL demands a significantly greater torsional moment to achieve the same total ROM as one with a lower NFL (22). As mentioned above, the two-level and three-level models only need 0.41 Nm–0.72 Nm and 0.66 Nm–0.97 Nm, respectively, to achieve the same ROM as the four-level model (1.5 Nm). Therefore, due to a greater moment load, a greater NFL induces greater compensation for the ROM and greater disc stress and FJF in the adjacent segment, which may facilitate the progression of disc injury and, ultimately, the development of ASD.

Another important finding is that in ACAF, ASD is more likely to occur in the upper adjacent segment than in the lower segment. As shown in the results, the maximum differences between the upper and lower adjacent segments across the three ACAF models were 39%–56% for mean disc stress and 70.35–93.39% for FJF. One possible explanation for this phenomenon is that the disc size gradually increases from C2/3 to C7/T1. As shown in our previous study, the cross-sectional area of the intervertebral disc gradually increases from 211 mm^2^ to 426 mm^2^ when moving from the C2/3 segment to the C7/T1 segment (13). Additionally, another anatomical study by our group based on CT images revealed that as the segment shifted from C3/4 to C6/7, the anteroposterior and transverse diameters of the intervertebral disc also increased progressively from 15.76 mm and 15.1 mm to 16.83 mm and 20 mm, respectively (24). Therefore, under the same follower load, the upper adjacent disc incurs significantly higher stresses than the lower adjacent disc does, primarily because of its smaller size. Furthermore, a smaller disc size reduces its bending stiffness, which in turn leads to greater deformation and higher stress when subjected to the same torsional moment.

While clinical evidence supporting the relationship between the NFL and ASD in patients with ACAF remains limited, the incidence of ASD increases with increasing NFL in other cervical fusion procedures. ASD is a common complication of cervical fusion procedures, and approximately 5.78% of cervical fusion patients require revision surgeries due to ASD (25). Shin (26) conducted a retrospective cohort study to investigate the incidence of ASD in patients who underwent anterior cervical discectomy and fusion (ACDF); after a follow-up of 31.9 months, the incidence of ASD was 15.38% for one-level ACDF, 28.57% for two-level ACDF, and 39.47% for three-level ACDF (26). Additionally, compared with the lower adjacent disc, the upper adjacent disc exhibited a greater ROM, making it more prone to developing ASD (26). Similarly, Hins et al. (27) retrospectively investigated the clinical outcomes of 369 patients who underwent posterior cervical decompression and fusion. They found that a shorter fusion level (C3–C6) was associated with a significantly lower incidence of symptomatic pseudarthrosis or ASD (2.8%) than a longer fusion level was (C3–C7, 8.3%) (27). These findings further support our hypothesis that the NFL may influence the progression of ASD in patients with ACAF by altering the biomechanical properties of the cervical spine, providing indirect evidence that the NFL is a risk factor for ASD in patients with ACAF.

### Surgical segment

Our results further demonstrated that increasing the NFL increases intervertebral space micromotion, which potentially increases the risk of pseudarthrosis following ACAF. As illustrated in Figure 3, the ROM at the surgical segment increased from 0.03°–0.19° to 0.29°–0.51° as the NFL increased from two levels to four levels. Additionally, the ROM for each single fusion segment increased from 0.01°–0.11° to 0.04°–0.2°. Ledet et al. (28) reported that suitable micromotion at the surgical level provides appropriate mechanical stimulation to induce the osteoblastic differentiation of mesenchymal stem cells within the intervertebral space, thereby accelerating osteogenesis and facilitating solid bony fusion. In contrast, insufficient motion at the surgical segment leads to stress shielding, whereas excessive motion causes instability and ultimately results in pseudarthrosis (28). The mechanism underlying increased micromotion with a greater NFL is that the cervical spine requires greater torsional moments to achieve the same total ROM as a spine with fewer fusion levels (22). This greater movement induces greater deformation at the surgical segment, thereby increasing the micromotion of the intervertebral space. Our findings are consistent with those of previous clinical studies. White et al. (29) retrospectively reviewed the records of 63 patients who underwent four-level ACDF and reported that 37 patients (58.7%) developed radiographic pseudarthrosis at a mean follow-up of 2.6 years, with a mean nonfused level of 1.35. Choi et al. (30) analyzed 84 consecutive patients who underwent ACDF and found that the pseudarthrosis rate was 22.4% for patients who underwent single-level ACDF compared with 50% for patients who underwent three-level ACDF at the final follow-up. Collectively, these findings suggest that increasing the NFL is a potential risk factor for pseudarthrosis in patients with ACAF.

Regarding cage subsidence, our results also showed that as the NFL increased from two levels to four levels, the mean stress of the endplate increased by approximately 98%–243% (Figure 6). This increase in mean endplate stress significantly increases the risk of endplate breakage and subsequently leads to cage subsidence in patients with ACAF. Additionally, as shown in Figure 8, the mean stresses of the plate, screw, and screw–bone interface increased by 23.37%–124% as the NFL increased from two levels to four levels. These factors significantly increase the risks of screw loosening, breakage, and shifting, subsequently leading to implant failure (8, 31). From the perspective of biomechanics, the main reason for these outcomes is the increased load on the surgical segment. As the NFL increases, the torsional moment needs to be increased to achieve a normal total ROM of the cervical spine (22). This change subsequently yields a greater load on the surgical segment and causes the endplate and screw‒plate system to experience larger deformations and stresses, which increase the likelihood of fatigue damage and eventually develop into cage subsidence and implant failure (32, 33).

Previous clinical studies also revealed relationships between the NFL and cage subsidence and implant failure in patients who underwent other types of cervical fusion surgeries, which further suggests that the NFL is a risk factor for cage subsidence and implant failure in patients treated with ACAF. In a clinical study conducted by Doi et al. (34), they used multivariate logistic analysis based on 49 patients to investigate the risk factors for cage subsidence in patients treated with ACDF, including cage size, endplate geometry, surgical level, and NFL. The results demonstrated that the NFL was the only risk factor for cage subsidence (34). Similarly, Kao et al. (35) investigated the clinical outcomes of 82 patients who underwent ACDF. The results revealed that a greater NFL was associated with a higher rate of cage subsidence than one or two fusion levels were (*P* = 0.038) (35). Song et al. (31) retrospectively investigated the incidence of hardware complications, including cage subsidence, plate looseness, and screw looseness and breakage, in 808 patients who underwent ACDF. They reported that the rate of hardware complications increased as the NFL increased, with 6.3% for one-level fusion, 18.8% for two-level fusion, 30.2% for three-level fusion, and 29.5% for four-level fusion (31).

### ACAF vs. ACDF: distinct biomechanical responses to NFL

Although ACDF also has a higher ASD risk in the upper adjacent segment (26), the biomechanical responses to increased NFL are fundamentally distinct in ACAF, which confirms the novelty of this study rather than a simple extension of ACDF conclusions. In ACDF, upper segment vulnerability is merely a passive compensation for fusion segment motion loss (26). In contrast, the pronounced upper adjacent segment hyper-compensation in ACAF is a synergistic result of its unique vertebral body-OPLL complex anterior translation and the smaller anatomical size of upper cervical discs (24), leading to far more dramatic changes in ROM (52.6%–109.5%), disc stress (38.46%–46.15%) and FJF (0%–116.5%) than in ACDF, with the upper and lower adjacent segment FJF difference even reaching 70.35%–93.39%. Additionally, ACAF exhibits two specific biomechanical changes unreported in ACDF: a nonlinear increase in surgical segment micromotion (0.03°–0.19° in two-level to 0.29°–0.51° in four-level) and a sharp concentration of endplate stress (98%–243% increase in four-level fusion). These changes are directly caused by ACAF-specific procedures including vertebral osteotomy and uncinate process grooving, which alter the cervical load-bearing axis and structural rigidity more extensively than simple discectomy in ACDF. Collectively, our study clarifies the ACAF-specific biomechanical pattern between NFL and postoperative complications, filling the critical research gap in ACAF surgical planning.

### Future prospects

This study has inherent limitations. It relies on FE simulation, which uses idealized cervical anatomical models and simplified loading protocols. These methods cannot fully capture the complexity *in vivo*, such as dynamic muscle forces, individual bone quality variations or postoperative tissue remodeling. While clinical evidence from other cervical fusion procedures, such as ACDF and posterior cervical fusion, supports the link between the NFL and long-term complications and reinforces our simulation-derived trends, direct validation from clinical trials of ACAF patients remains essential (21, 26, 27, 29–34).

Future strategies could involve personalized preoperative fusion-level planning to address the increased risk of ASD with more fusion levels. This planning should be based on patient-specific disc size and alignment to avoid unnecessary segment extension (36). Dynamic fixation or hybrid constructs that combine fusion with disc replacement can evenly distribute cervical motion, and postoperative neck muscle rehabilitation can increase stability (37, 38). With respect to cage subsidence and implant failure, optimizing intraoperative endplate preparation to preserve integrity and reduce load concentration is a promising approach (39). High-strength, biomechanically matched implants such as porous cages that promote better bony integration should be used, and preoperative bone mineral density assessments should be conducted to tailor implant selection (40, 41). These improvements, paired with clinical validation, will increase the translational value of our biomechanical insights for ACAF surgical practice.

Notably, the present FE model applied a “tie” constraint to all implant-bone and implant-implant interfaces (cage–endplate, screw–bone, screw–plate) to simulate ideal rigid fixation conditions, which neglected the potential micro-loosening, interface slip and stress shielding at the fixation interfaces that commonly occur in clinical practice (31, 41). Such *in vivo* alterations in fixation stability can alter the actual stress distribution at the surgical segment and further elevate the risk of implant-related complications including screw loosening, breakage and cage subsidence (7, 31). In clinical settings, fixation failure is also closely associated with individual variations in bone mineral density and intraoperative fixation technique (41), which were not incorporated into this idealized FE model, potentially leading to a slight overestimation of the mechanical stability of the ACAF construct.

## Conclusions

This study employed finite element analysis to investigate the influence of the NFL on the long-term complications of ACAF with two-level, three-level, and four-level ACAF models. The results indicated that a greater NFL more significantly altered the biomechanical properties of the cervical spine, increasing the ROM, disc stress, and FJF in adjacent segments, with the upper adjacent segment showing the most notable changes; in turn, these changes collectively increased the risk of ASD. A greater NFL also increased the ROM of the surgical segment, which in turn increased micromotion in the fusion space and therefore the risk of pseudarthrosis. Additionally, it amplified stress on the endplate, fixation plate, screws, and screw–bone interface, leading to increased incidences of cage subsidence and implant failure.

These findings confirm that the NFL is a potential risk factor for long-term complications following ACAF and provide valuable ACAF-specific mechanism-based biomechanical evidence for optimizing surgical planning for ACAF, such as by refining the NFL selected and improving implant design, to mitigate complication risks. While the conclusions are supported by clinical data from other cervical fusion procedures, future prospective clinical trials specific to ACAF are necessary to validate these simulation-derived insights. This validation will ultimately contribute to more effective and safer ACAF surgical practices for treating cervical OPLL.

## Data Availability

The raw data supporting the conclusions of this article will be made available by the authors, without undue reservation.
